# Assessment of alternative techniques to quantify the effect of injury on soft tissue in closed ankle and pilon fractures

**DOI:** 10.1371/journal.pone.0268359

**Published:** 2022-05-11

**Authors:** Sascha Halvachizadeh, Roman Pfeifer, Yannik Kalbas, Simone Schuerle, Paolo Cinelli, Hans-Christoph Pape

**Affiliations:** 1 Department of Trauma, University Hospital Zurich, Raemistrasse, Zurich, Switzerland; 2 Harald Tscherne Laboratory, University of Zurich, Sternwartstrasse, Zurich, Switzerland; 3 Institute of Translational Medicine, Department of Health Science & Technology, ETH Zurich, Zurich, Switzerland; John Hunter Hospital and University of Newcastle, AUSTRALIA

## Abstract

**Introduction:**

Local soft tissue status (STS) guides the timing for definitive surgical treatment strategies of fracture fixation around the ankle joint. The aim of this study was to assess different types of new technical devices in relation to the surgical treatment in closed ankle and pilon fractures.

**Methods:**

This study was designed as a cohort study. Adult patients admitted between February 1, 2019 and December 31, 2020 presenting with closed ankle fracture requiring surgical treatment were eligible. The exclusion criteria were previous injuries to the lower extremity, acute deep venous thrombosis, skin diseases, and delayed presentation (admission >24 hours after injury). Moderate-energy trauma includes injuries sustained during team sports, biking, and running. The primary outcome was the assessment of the degree of soft tissue involvement following closed fractures by comparing different techniques focusing on the ankle region and including ankle and pilon fractures. The variables of interest included the circumference of soft tissue swelling around the ankle, determined within a 5-mm range in the area of the medial and lateral malleolus and the bone-skin distance on a plain radiograph, determined by the largest distance from the malleolus to the border of the soft-tissue shadow. STS assessment included optical measures of local perfusion (O2C, Lea Inc. Germany) and tactile measures of mechanical characteristics (Myoton^®^ tensiometer AS, Estonia). Measurements of Group Temp (temporary stabilization) and Group Def (definitive surgery) were taken on admission and prior to the treatment strategy decision. The contralateral non-injured ankle served as a control. The quality of assessment tools was quantified by calculating the smallest detectable change (SDC).

**Results:**

In total, 38 patients with a mean age of 40.4 (SD 17.8) years were included. The SDC was 3.2% (95%CI 2.5 to 3.8) for local blood flow and 1.1% (95%CI 0.4 to 1.7) for soft tissue stiffness. The circumference of the injured area at admission was significantly higher than that of the healthy site (28.2 [SD 3.4] cm versus 23.9 [SD 2.4] cm, p < 0.001). The local perfusion (blood flow 107.5 (SD 40.79 A.U. vs. 80.1 [SD 13.8] A.U., p = 0.009), and local dynamic stiffness of the skin (668.1 (SD 148.0) N/m vs 449.5 (SD 87.7) N/m, p < 0.001) were significantly higher at the injured site. In Group Temp, the local blood flow was significantly higher when compared with Group Def (109.6 [SD 39.8] vs. 94.5 [SD 13.0], p = 0.023). The dynamic stiffness of the soft tissue was significantly higher in Group Temp (679.4 N/m [SD 147.0] N/m vs. 573.0 N/m (SD 93.8) N/m, p < 0.001). The physical properties of STS were comparable among the fracture types. None of the included patients had local soft tissue complications.

**Conclusion:**

Closed fractures of the ankle and the pilon are associated with an increase in local circulation and local soft tissue stiffness and tension. These changes of the STS following injury can be quantified in a standardized and reproducible manner.

## Introduction

Local soft tissue status (STS) is amongst the most relevant predictors of complications [1] and therefore guides the surgical treatment strategy for fractures [2]. STS assessment in open fractures is based on clinically well-established methods [3, 4]; however, in closed fractures, it is much vaguer and still subject to the examiner’s expertise and the surgeon’s experience. To approximate some degree of standardized measure, the severity of soft tissue damage is estimated with measures of energy, fracture classification [5], or subjective clinical examination [6] (inspection, palpation [e.g., the simple “wrinkle sign”], and circumference measurement). Soft tissue classifications currently in use have a low interobserver agreement in closed (0.65 to 0.81) [7] and open (0.44) fractures [8], indicating the necessity of novel assessment methods for STS in closed fractures. Following trauma, the soft tissue experiences mechanical stress that leads to alteration of the fibrous structural features that serves as a direct measure of its health and functionality [9]. Further, mechanical stress with alterations of connective tissue disturbs perfusion and alters tissue viscoelasticity [10]. These effects lead to increased perfusion, soft tissue swelling and increased surface tension. In delayed presentations and other special situations, fracture blisters may develop due to the sustained tissue injury or to lack of fixation by direct or indirect measures. Recently, new technical options have become available that measure the local circulation based on optical sensors [11]. In a similar fashion, tactile measures are available to quantify skin stiffness [12], which may also be useful in the quantification of STS or may help to provide a risk profile for tissue circulation. Therefore, this study aimed to measure different aspects of STS caused by acute fractures in order to test our hypothesis that changes in STS can be quantified with tissue perfusion and tissue tension.

## Methods

### Ethical review committee statement

This prospective cohort study was approved by the institutional review board (IRB) and the local ethics committee (Nr.: 2018–01489) and was conducted in accordance with the ethical standards in the 1964 Declaration of Helsinki. All included patients were required to sign an informed consent form prior to inclusion.

Result reporting followed the criteria for "Strengthening the Reporting of Observational Studies in Epidemiology" (STROBE) [13]. Patients with isolated closed ankle fractures admitted for surgical treatment were eligible for inclusion. All patients were assessed for eligibility by a study investigator, who was not part of the patient’s medical treatment. Eligible patients were informed about the study and their inclusion and exclusion criteria reviewed. Recruitment took place between February 1, 2018 and December 31, 2020.

### Participants: Inclusion and exclusion criteria

Patients were included if they fulfilled the following criteria: isolated closed ankle fracture (Danis-Weber Type B or Type C, trimalleolar fractures, as defined by lateral and medial malleolus fracture and Volkmann fracture, or pilon fractures); diagnosis and treatment on the day of injury; surgical fracture fixation; and age 18 years and older.

The general exclusion criteria were previous injuries to the lower extremities (including soft tissue injuries), a bilateral fracture pattern, previous deep vein thrombosis (DVT), and skin diseases affecting the surrounding soft tissue envelope of the fracture.

### Outcome and definition

The primary outcome was the degree of soft tissue involvement following closed fractures, which was assessed by comparing different techniques with a focus on the ankle region and including ankle and pilon fractures. The soft tissue assessment included two measures that potentially affect the surgical treatment strategy. First, the local circulation has been reported to represent a risk factor for deep surgical infection [14]. The local circulation is affected by injury [15] and has been utilized as a measure of the vitality of soft tissue flaps [16]. Second, local biomechanical soft tissue properties may influence the treatment strategy [17].

Local complications were assessed during hospitalization and during the follow-up period and included superficial and deep infections, wound dehiscence, and the requirement of temporal soft tissue coverage. Local complications were evaluated by an interdisciplinary approach including the treating surgeon, an infectious diseases specialist, and a reconstructive surgeon [18].

### Local perfusion

The “Oxygen to see” device (O_2_C, Lea Germany) assesses the local perfusion of intact skin [19] by using various optical sensors to measure local blood flow, oxygen saturation, and the relative hemoglobin amount [11]. O_2_C measurements were performed at the soft spot between the lateral malleolus and the Achilles tendon.

### Local biomechanical soft tissue properties

The digital palpation device calculates tension with damped natural oscillation [20]. It measures surface tension, as well as other biomechanical and viscoelastic properties, by applying a light force to the skin. Measurements provide frequency (Freq. [Hz]), stiffness (stiff. [N/m]), and stress-relaxation time (S/R, [ms]) by tactile measures of the skin.

### Clinical measures of soft tissue status

Four different techniques for assessing STS were compared: 1.) Radiologic assessments and measurement of bone-skin distance, 2.) Circumference measurements utilizing measuring tape [21], 3.) Local perfusion utilizing the oxygen to see device (Lea Inc., Germany O_2_C), and 4.) Local tension utilizing the digital palpation device (Myoton AS, Estonia).

### Radiologic assessment and circumference

From routine X-ray or CT scans, the bone-to-skin distance was measured from the most lateral aspect of the lateral malleolus [mm]. The circumference was measured around the medial and lateral malleolus, representing a standard ankle circumference [cm].

### Measurement protocol

Measurements were taken on the injured side; the non-injured (healthy) side served as control. Measurements were performed simultaneously on predefined areas (soft spot between the lateral malleolus and Achilles tendon for perfusion and tension; around the medial and lateral malleolus for circumference). Measurements were taken on the day of the injury (at admission) and the day of definitive surgery prior to the surgery in the preparation room (pre-op).

### Clinical treatment protocol and treatment strategy

According to our institutional protocol, local STS is assessed by at least one attending surgeon. This assessment includes a clinical examination of the skin, local senso-motoric status, and the amount of swelling. Fractures in patients awaiting definitive surgery following the assessment of STS were temporarily stabilized with either open cast stabilization (splint, bi-valved, or cut-away cast) or external fixation (Group Temp). All patients remained in hospital and were assessed on daily a basis for surgery by the treating trauma surgeon, following our institutional protocol. Definitive surgery was performed with plate osteosynthesis in the usual manner (Group Def). The treatment strategy was defined by the treating surgeon, and the examiner and patient were blinded to the intended strategy during measurements. The follow-up period encompassed every routine clinical follow-up visit for 6 months (6 weeks, 3 months, and 6 months after definitive surgery).

Comorbidities are summarized according to the Charlson Comorbidity Index (CCI) [22]. Smokers were defined as currently active smokers and patients who had quit smoking within the past 5 years. Substance abuse included current alcohol abuse (as defined by self-reported consumption of at least five alcoholic beverages per day) [23, 24], intravenous drug use (IVDU), or the current regular use of other illicit substances. Diabetes included Type 1 and Type 2 diabetes. Type 2 diabetes was further stratified according to insulin dependence. Skin diseases included chronic and acute skin diseases such as psoriasis, skin infection, and dermatitis. Local soft tissue complications included superficial and deep infections, wound dehiscence, or the requirement of temporary soft tissue coverage.

### Interobserver variability

Physicians performed the same measurements independently of each other on one uninjured representative ankle. The measurement results of each examiner were collected and the smallest detectable change (SDC) for each measurement technique calculated to assess interobserver variability. The SDC is the smallest change that is required for recognition by the measuring device. A lower SDC represents a more sensitive measurement technique.

### Statistical analysis

Data were collected prospectively using “Research electronic data capture” (REDCap^®^). Continuous variables are displayed as means with standard deviations (SD), and categorical variables as counts with percentages. Measurement error was defined as the systematic and random error of a measurement that is not attributed to true changes in the measured construct [26]. Repeated measurements of the non-fractured site were used to determine the measurement error. We assumed that there would be no relevant change in the measurements. Measurement error is expressed as the standard error of measurement (SEM). The SDC represents the minimal change that a patient must show on the scale to ensure that the observed change is real and not just measurement error. The SDC was calculated as $1.96\;\times \sqrt{2}\;\times SEM$, and the confidence interval (CI) was calculated as $SDC\;\pm 1.96\;\times \frac{2}{\sqrt{2}}$ [22]. To increase comparability across measurement devices, the percentage SDC and the 95%CI were calculated. To check for normality, the distributions of variables were visualized using QQ-plot and a histogram and tested using the Shaprio test. For local soft tissue circulation measurements, each assessment was performed for 15 seconds, and the median of these measures was calculated and documented. For local soft tissue tension, each assessment was performed five times per measurement, and the means of these measures were calculated and documented.

Parametric tests were utilized for comparison of normally distributed variables, non-parametric tests for skewed variables. Group comparisons were performed using Student’s t-test. To assess the impact of fracture morphology, soft tissue measurements were compared using ANOVA. The dynamics of soft tissue injury were assessed using dependent t-tests within the measurement. The statistical significance level was set at p ≤ 0.05. All statistical analyses were performed using R (R Core Team, 2020. R: A language and environment for statistical computing. R Foundation for Statistical Computing, Vienna, Austria. URL https://www.R-project.org/).

## Results

### Study population

**Fig 1:**
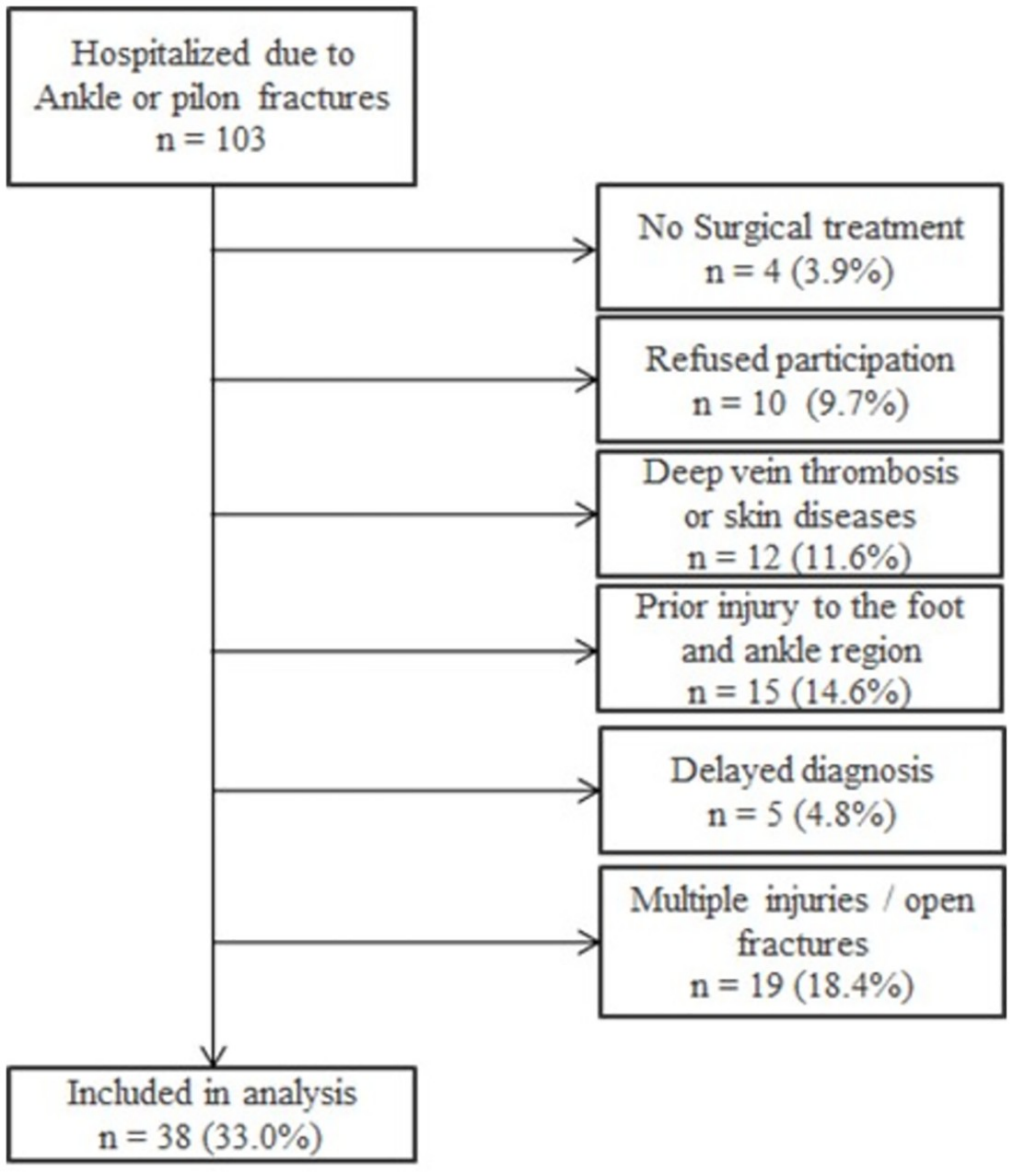
Flow chart of included and excluded patients.

During the recruitment period, 103 patients were hospitalized due to an ankle or pilon fracture. Out of these 34 (33.0%) were included in this analysis (Fig 1). The included patients had an average age of 40.4 (SD 17.8) years; 18 (47.4%) were female. The average BMI was 24.5 (SD 5.7) kg/m^2^. Eleven (28.9%) patients were active smokers. Most patients had suffered an isolated lateral malleolus fracture (n = 15, 40.5%), followed by trimalleolar fractures (n = 11, 28.9%), and pilon fractures (n = 4, 10.5%) (Table 1).

**Table 1:** Patient demographics.

n	38
Age [years], mean (SD)	40.4 (17.8)
Female gender, n (%)	18 (47.4)
BMI [kg/m2], mean (SD)	24.5 (5.7)
Diabetes, n (%)	2 (5.2)
PAD, n (%)	1 (2.6)
Smoking, n (%)	11 (28.9)
Substance abuse, n (%)	3 (7.9)
CCI [points], mean (SD)	0.9 (1.9)
Type of ankle fracture, n (%)	
Danis-Weber B/C	25 (65.8)
Trimalleolar	9 (23.7)
Pilon	4 (10.5)

n = Number; SD = Standard deviation; BMI = Body mass index; PAD = Peripheral artery disease; CCI = Charlson comorbidity index.

### The effect of injury on local soft tissue

On admission, the circumference of the injured site was 17.9% greater than that of the not-injured contralateral site (p ≤ 0.001). The bone-skin distance at the injured site was 2.1 times the distance at the healthy contralateral site (p ≤ 0.001). Local perfusion was significantly higher after the injury: blood flow was increased by 34.2% (p = 0.089), local hemoglobin by 101.4% (p ≤ 0.001), and local oxygenation by 19.3% (p = 0.021). The local tension of the soft tissue was further affected by the injury: the local frequency was increased by 27.5% (p ≤ 0.001), local stiffness increased by 48.6% (p ≤ 0.001), and the S/R time decreased by 28.1% (p ≤ 0.001) (Table 2).

**Table 2:** Quantification of physical soft tissue properties at admission.

		Injured	Non-injured	95% CI	p-value
Clinical measure	Circumference [cm], mean (SD)	28.2 (3.4)	23.9 (2.4)	2.4 to 6.2	<0.001
X-Ray	Bone-skin distance [mm], mean (SD)	9.7 (3.8)	4.7 (3.0)	3.4 to 4.7	<0.001
Local perfusion (O2C)	Blood flow [A.U.], mean (SD)	107.5 (40.7)	80.1 (13.8)	7.6 to 47.3	0.008
	Local hemoglobin [A.U.], mean (SD)	72.5 (24.0)	36.0 (7.9)	24.8 to 48.2	<0.001
	Local oxygenation [A.U.], mean (SD)	71.7 (17.9)	60.1 (11.9)	1.9 to 21.4	0.021
Local tension (Myoton)	Frequency [Hz], mean (SD)	26.9 (5.1)	21.1 (1.9)	3.4 to 8.4	<0.001
	Stiffness [N/m], mean (SD)	668.1 (148.0)	449.5 (78.7)	141.9 to 295.3	<0.001
	S/R time [ms], mean (SD)	8.7 (2.0)	12.1 (1.7)	8.7 to 12.1	<0.001

cm = Centimeter; mm = Millimeter; ms = Milliseconds; A.U. = Arbitrary unit; SD = Standard deviation; Hz = Hertz; N/m = Newton/meter; CI = Confidence interval.

### Physical properties of STS in Group Temp versus Group Def

The mean time from injury to definitive surgery was 3.8 (SD 2.2) days, and the median time from injury to definitive surgery was 4 days (IQR 2.75). The soft tissue circumference measurements in Group Temp were 8.0% higher than those in the definitive surgery group (statistically not significant, p = 0.153). The local blood flow was 13.8% lower in Group Def when compared with Group Temp (p = 0.023), and the local hemoglobin amount remained comparable (p = 0.808). The local oxygenation was 3.0% lower in Group DEF (p = 0.08). In Group Temp, the local soft tissue frequency was 20.9% higher than that of patients prepared for definitive treatment (p < 0.001). The soft tissue stiffness was 15.6% higher (p < 0.001), and the stress-relaxation time was 13.3% lower in Group Temp vs Group Def (p = 0.043) (Table 3).

**Table 3:** Objective soft tissue characteristics prior to surgical management.

		Group Temp	Group Def	p-value
Clinical measure	Circumference [cm], mean (SD)	28.6 (3.6)	26.3 (4.6)	0.153
Local perfusion (O2C)	Blood flow [A.U.], mean (SD)	109.6 (39.8)	94.5 (13.0)	0.023
	Local hemoglobin [A.U.], mean (SD)	72.3 (24.2)	72.6 (29.7)	0.808
	Local oxygenation [A.U.], mean (SD)	71.7 (17.0)	69.5 (16.7)	0.08
Local tension (Myoton)	Frequency [Hz], mean (SD)	27.2 (4.9)	22.5 (4.3)	<0.001
	Stiffness [N/m], mean (SD)	679.4 (147.0)	573.3 (93.8)	<0.001
	S/R time [ms], mean (SD)	8.5 (1.9)	9.8 (1.6)	0.043

cm = Centimeter; mm = Millimeter; ms = Milliseconds; A.U. = Arbitrary unit; SD = Standard deviation; Hz = Hertz; N/m = Newton/meter.

### Interobserver variability: SDC

The assessment of circumference required that examiners detect a minimum change of 3.4% (95%CI 2.8 to 4.1). The assessment of radiologic bone-skin distance required that the examiners detect a minimum change of 5.4% (95% CI 4.7 to 6.0). The SDC for local blood flow was 3.2% (95%CI 2.5 to 3.8), for local hemoglobin amount 2.1% (95%CI 1.4 to 2.7), and for local oxygenation 2.4% (95%CI 1.7 to 3.0). The SDC for frequency was 2.0% (95%CI 1.4 to 2.7), for stiffness 1.1% (95%CI 0.4 to 1.7), and for S/R-time 1.4% (95%CI 0.7 to 2.0%).

### Impact of fracture classification on local soft tissue damage

Fifteen (40.5%) patients suffered an isolated lateral malleolar fracture, 11 (28.9%) a trimalleolar fracture, and 4 (10.5%) a pilon fracture. Both the circumference and the bone-skin distance measurements were comparable in these groups (p = 0.675). The local blood flow was comparably lower in isolated lateral malleolar fractures (p = 0.062). The local hemoglobin amount was comparable (p = 0.075). The measures of local tension were comparable among fracture types (p = 0.745).

### The effect of comorbidities on physical properties of STS

In total, 24 (63.2%) patients had a CCI of 0 points (healthy), and 14 (36.8%) had a CCI of one point or higher (range 1 to 8 points, median 1 point, mean 2.6, SD 2.5 points). The clinical, local perfusion, and local tension measurements were comparable in these groups (Table 4). Patients who smoked had a significantly lower stress-relaxation time at the injured ankle when compared with non-smokers (7.5 [SD 2.0] ms vs. 9.4 [SD 1.6] ms, 95%CI 0.1 to 3.8, p = 0.037).

**Table 4:** Measurement of the soft tissue status in closed ankle or pilon fractures stratified according to comorbidities.

		Healthy n = 24	Comorbidities n = 14	p-value
Clinical measure	Circumference [cm], mean (SD)	27.6 (3.3)	29.2 (3.7)	0.323
X-Ray	Bone-skin distance [mm], mean (SD)	9.9 (3.3)	9.5 (4.7)	0.759
Local perfusion (O2C)	Blood flow [A.U.], mean (SD)	99.4 (46.9)	122.7 (20.8)	0.231
	Local hemoglobin [A.U.], mean (SD)	73.1 (24.5)	71.4 (24.9)	0.885
	Local oxygenation [A.U.], mean (SD)	71.0 (24.5)	73.1 (15.7)	0.801
Local tension (Myoton)	Frequency [Hz], mean (SD)	25.6 (4.4)	29.4 (5.8)	0.120
	Stiffness [N/m], mean (SD)	628.5 (141.2)	741.6 (140.6)	0.104
	S/R time [ms], mean (SD)	9.1 (1.9)	7.8 (2.0)	0.187

cm = Centimeter; mm = Millimeter; ms = Milliseconds; A.U. = Arbitrary unit; SD = Standard deviation; Hz = Hertz; N/m = Newton/meter.

### Local soft tissue complications

All of the 38 included patients completed the 6-week and the 3-month follow-up. One patient did not complete the 6-month follow-up. All 38 patients were included in the analyses of primary and secondary outcomes. In total, 7 patients (18.4%) required a syndesmosis stabilization and underwent out-patient removal of the syndesmosis screws 6 weeks after surgery. None of the patients who completed the follow-up had local soft tissue complications or required prolonged antibiotic treatment.

## Discussion

Ankle fractures account for 9% of all fractures and 40% require surgical management [25]. The severity of local soft tissue damage represents a sustained problem associated with unplanned revision surgeries and might increase the duration of hospital stay from a mean of 4.4 days to 11.8 days [26]. Severe soft tissue damage leads to 10-fold higher direct costs when compared with non-displaced ankle fractures with minimal soft tissue damage ($79-$268 versus $2,860-$19,555) [27]. STS guides treatment and predicts the outcome following fractures of the extremities [28]. This preliminary study aimed to associate the physical properties of injured STS following closed ankle fractures with the current treatment strategy and found that the investigated techniques measured an increased circulation and increased tension in injured soft tissue in closed fractures. The effect of trauma on soft tissues in closed fractures can be quantified in a reproducible and standardized manner.

It is well known that the trauma energy affects both the bone and the surrounding soft tissue. While routine radiological measures are widely utilized to classify fractures, STS is only addressed marginally. The techniques evaluated in this study can quantify the effect of trauma on the STS. It reacts with a cascade of biological and biomechanical changes affecting the local circulation, inflammation, immune reaction, and protective properties [29–31]. This includes marked hemorrhage and edema [32], disruption of the microvascular system [33], and stimulation of further inflammatory responses [34]. These tissue reactions increase the risk for complications, surgical site infections, and impaired wound healing and have led to the development of staged management and soft-tissue-guided treatment strategies [35]. The increased hemorrhage and increased blood flow were measured with the optical sensor, and the biomechanical changes in terms of tissue tension following soft tissue swelling were quantified with tactile measures.

The timing of definitive surgical treatment of fractures is associated with the time-dependent release of cytokines and complications [36]. The healing process is a complex interaction of local and systemic factors and is initiated by the release of local mediators from soft tissue [37]. These early dynamic changes are represented in the local STS, and the presented measurement techniques might quantify the results of the healing process: local swelling and the associated local tension decreases, as does the local blood flow. The effects of these changes might support the choice of an optimal timepoint for definitive surgery while decreasing the risk of local complications [38]. The interobserver variability of the novel measurement devices is in accordance with the literature [11, 12, 39]. The measurement of swelling utilizing measurement tape represents the circumference, but not the tension or blood circulation, and is more observer dependent when compared with tactile measures. When compared with routine clinical measures, the decreased interobserver variability indicates an improved and reproducible objective quantification method for local STS.

In our series, the degree of soft tissue damage is not associated with the fracture classification. This is surprising at first glance, as trauma energy is discussed as a cofactor in soft tissue damage [5]. For fracture classification, however, trauma energy and trauma mechanisms are poor predictors [40]. Further, preexisting conditions that result in lower bone quality (e.g., osteoporosis) might result in more severe fractures after lower energy trauma [41]. In contrast to fracture classification, soft tissue damage presents with dynamic properties [42].

### Strengths and limitations

This is the first study that assesses the implementation of these novel devices in closed extremity fractures. Certain comorbidities that might affect the local soft tissue were not taken into consideration. In order to minimize this potential bias, the study protocol and the analysis of the present study compared the injured side with the healthy side in each patient and therefore controlled for between-patient variability.

One might suggest that the clinical measurements provided in this study are not routinely used. The clinical application and feasibility of the presented new techniques must be evaluated in a larger series. This study presents innovative quantification methods for STS in closed fractures.

The interobserver variability is currently unknown. The calculation of the SDC does not represent a common assessment of interobserver variability. However, to calculate Cohen’s kappa, a reference test is required [15]. Based on the lack of standardized and reproducible quantification methods for soft tissue damage, the calculation of Cohen’s kappa is not feasible.

Comorbidities, especially diabetes and smoking, are well known risk factors for the development of local soft tissue complications. While these factors were evaluated, they were not included in the present study based on the potential for type 2 errors. Local STS, however, might be a more relevant guide for the treatment strategy when compared with comorbidities. Surprisingly, none of the patients developed complications after 3 and 6 months. An explanation may lie in the fact that all of these patients were primarily admitted to us, and no patient with a delayed presentation was included in this pilot study. The complication rate was not the primary outcome of the present study, which is underpowered to calculate associations of these novel measurements and the incidence of local soft tissue complications. We still believe that these measurements have merit to support clinical decision making on the timing of definitive fracture fixations.

## Conclusion

Closed fractures of the ankle and pilon are associated with an increase in local blood flow and soft tissue stiffness. The investigated new devices were able to detect changes of the local STS in a standardized and reproducible manner.
